# Etymologia: Glanders

**DOI:** 10.3201/eid2101.ET2101

**Published:** 2015-01

**Keywords:** etymologia, glanders, Burkholderia mallei, farcy

## Glanders [glanʹdərz]

From the Old French *glandres* (“glands”) describing the enlargement of the parotid or submaxillary lymph nodes that is pathognomonic of the disease, glanders is a contagious disease of horses. Glanders is caused by *Burkholderia mallei* and is communicable to humans but should not be confused with human melioidosis, caused by *Burkholderia pseudomallei*. The chronic, cutaneous form of glanders presents as ulcerated skin lesions along major lymph and blood vessels and is known as farcy (from the Latin *farcire*, “sausage”). Among the first descriptions of glanders is in the writings of Aristotle: “The ass suffers chiefly from one particular disease which they call ‘melis.’” In later writings, “melis” became “malleus,” which became a generic term for epizootics. Glanders has been eliminated in many industrialized countries, including in the United States, where there have been no naturally acquired human or animal cases since World War II. More recently, glanders has been reemerging in parts of the world; since 2000, outbreaks in horses have been reported in North Africa, the Middle East, and other areas. 
